# Establishment and Implementation of Potential Fluid Therapy Balance Strategies for ICU Sepsis Patients Based on Reinforcement Learning

**DOI:** 10.3389/fmed.2022.766447

**Published:** 2022-04-14

**Authors:** Longxiang Su, Yansheng Li, Shengjun Liu, Siqi Zhang, Xiang Zhou, Li Weng, Mingliang Su, Bin Du, Weiguo Zhu, Yun Long

**Affiliations:** ^1^Department of Critical Care Medicine, State Key Laboratory of Complex Severe and Rare Diseases, Peking Union Medical College Hospital, Peking Union Medical College, Chinese Academy of Medical Sciences, Beijing, China; ^2^DHC Mediway Technology Co., Ltd., Beijing, China; ^3^Medical Intensive Care Unit, State Key Laboratory of Complex Severe and Rare Diseases, Peking Union Medical College Hospital, Chinese Academy of Medical Sciences and Peking Union Medical College, Beijing, China; ^4^Department of Information Center, State Key Laboratory of Complex Severe and Rare Diseases, Peking Union Medical College Hospital, Chinese Academy of Medical Sciences and Peking Union Medical College, Beijing, China

**Keywords:** sepsis, fluid therapy, machine learning, prognosis, model prediction

## Abstract

**Objective:**

Fluid therapy for sepsis patients has always been a problem that puzzles clinicians, that is, knowing when patients need fluid infusion and when they need negative fluid balance. Different clinicians may have different judgment criteria and make different decisions. Recently, studies have suggested that different fluid treatment strategies can cause different clinical outcomes. This study is intended to establish and verify a model for judging the direction of fluid therapy based on machine learning.

**Method:**

This study included 2705 sepsis patients from the Peking Union Medical College Hospital Intensive Care Medical Information System and Database (PICMISD) from January 2016 to April 2020. The training set and test set (January 2016 to June 2019) were randomly divided. Twenty-seven features were extracted for modeling, including 25 state features (bloc, vital sign, laboratory examination, blood gas assay and demographics), 1 action feature (fluid balance) and 1 outcome feature (ICU survival or death). SARSA was used to learn the data rules of the training set. Deep Q-learning (DQN) was used to learn the relationship between states and actions of the training set and predict the next balance. A double-robust estimator was used to evaluate the average expected reward of the test set in the deep Q-learning model. Lastly, we verified the difference between the predicted fluid therapy model and the actual treatment for the patient's prognoses, with sepsis patient data from July 2019 to April 2020 as the validation set.

**Results:**

The training set and test set were extracted from the same database, and the distribution of liquid balance was similar. Actions were divided into five intervals corresponding to 0–20, 20–40, 40–60, 60–80, and 80–100% percentiles of fluid balance. The higher the reward of *Q*(*s, a*) calculated by SARSA from the training set, the lower the mortality rate. Deep Q-learning indicates that both fluid balance differences that are too high and too low show an increase in mortality. The more consistent the fluid balance prediction with the real result, the lower the mortality rate. The smaller the difference between the prediction and the reality, the lower the mortality rate. The double-robust estimator shows that the model has satisfactory stability. The validation set indicates that the mortality rate of patients in the “predicted negative fluid balance and actual negative fluid balance” subgroup was the lowest, which was statistically significant, indicating that the model can be used for clinical verification.

**Conclusion:**

We used reinforcement learning to propose a possible prediction model for guiding the direction of fluid therapy for sepsis patients in the ICU. This model may accurately predict the best direction for fluid therapy, thereby improving patient prognosis.

## Introduction

Fluid resuscitation is the basic treatment for sepsis. However, in recent years, clinicians have found that an inappropriate infusion of large amounts of fluid may cause volume overload, which has become an independent risk factor for disability and death during critical illness (1–4). In the early treatment of sepsis, to improve organ perfusion, fluid therapy should be performed in a timely manner, but continuous positive fluid balance is not advocated. With the infusion of a large amount of fluid, a positive balance may cause treatment-related damage, such as capillary leak syndrome and pulmonary edema (5). In fact, an increasing number of studies suggest that having a negative fluid balance during the late treatment of sepsis can improve overall prognosis (6). However, how to prevent and address the fluid balance problem caused by volume therapy in clinical practice has become a problem that puzzles clinicians. Recently, physicians have used several methods to investigate resuscitation strategies for septic shock patients. Ma et al. (7) used finite mixture modeling and K-means clustering to identify subclasses of septic shock and dynamic treatment regime was used to give customized fluid volume and norepinephrine dose prescription for each patient. Lu et al. (8) explore the sensitivity of Dueling Double Deep Q-Networks to data preparation and modeling decisions in the context of hemodynamic management in septic patients. In this paper, we would like to explore fluid therapy balance strategies for ICU sepsis patients based on SARSA Algorithm, Q-learning and DQN model.

## Objects and Methods

### Patient Enrollment, Data Extraction, and Interventions

#### Inclusion and Exclusion Criteria

Patients who were diagnosed with sepsis between January 2016 and April 2020 at the Department of Intensive Care, Peking Union Medical College Hospital, were included in this study. Informed consent was given by patients (or their legally authorized representative/next of kin if the patients were dead) before any data extraction. Patients were excluded from this study if they met any of the following criteria: (1) they were younger than 18 years old or (2) they were admitted to the ICU for fewer than 24 h.

#### Definition of Sepsis and Initial Time of Fluid Resuscitation

The initial timing of fluid resuscitation is when patients are diagnosed as “sepsis”. Specifically, definition of sepsis is the time when both “infection is diagnosed” and “new onset of ΔSOFA ≥ 2” are reached (9). Infection was defined as follows: (1) if an antibiotic was used first, the etiology was obtained within 24 h after antibiotic treatment OR (2) if the etiology was noted first, the antibiotic was used within 72 h.

#### Data Extractions

Data were extracted from the Peking Union Medical College Hospital Intensive Care Medical Information System and Database (PICMISD). The parameters extracted from the database included demographics, vital sign data collected by bedside monitoring, laboratory examination data, blood gas analysis, microbiological examination results, antibiotic usage and total fluid balance. Notably, data de-identification was performed before any further analysis.

#### Ethical Approval

The authors assert that all procedures contributing to this work comply with the ethical standards of the relevant national and institutional committees on human experimentation and with the Helsinki Declaration of 1975, as revised in 2008. All the experimental protocols were approved by the Institutional Research and Ethics Committee of Peking Union Medical College Hospital, which approved this study for human subjects (No. SK-1241).

### Data Collection and Cleaning Strategy

**Figure 1:**
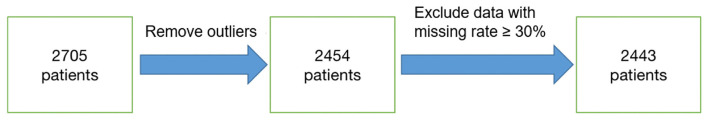
Data selection strategy.

A total of 2,705 patients were included based on sepsis 3.0. The average ICU time was 4.3-day, as calculated by PICMISD. We included patient data over each 6 h period up to 108 h (4.5 days), for 18 periods in total. For patients who transferred out of the ICU or died within 108 h, the last period was their last record in the ICU. The values of each feature are the average value for each period. The final outcome was patient death or survival over the last period, that is ICU survival or death. Death includes clinical death and the withdrawal of treatment without any further action. Each 6 h is identified as a block, which is abbreviated as “Bloc”. Because the laboratory features do not need to be measured frequently, the missing values were forward-filled. Missing values of other features with <30% missing rate were filled by KNN. Twenty-seven features were used for modeling, including 25 state features (bloc, vital sign, laboratory examination, blood gas and demographics), one action feature (fluid balance), and one outcome feature. We included 2,443 sepsis patients, with 2,095 survivors and 348 non-survivors. Each group of data includes all 27 features for one period for one patient. Each patient could contribute 18 groups of data at most depending on their ICU time. There were 31,425 groups of data generated from 2,443 patients (the data selection strategy is shown in Figure 1). The features and outlier manipulation criteria are shown in Table 1.

**Table 1:** Missing rate and outlier manipulation criteria of the modeling features.

**Feature**	**Description**	**Missing rate**	**Outlier manipulation method**
Invasive mean pressure (mmHg)	Vital sign	0.033	Exclude data with values below 0 and the value is 0 but not dead
Invasive systolic blood pressure (mmHg)	Vital sign	0.034	Exclude data with values below 0 and the value is 0 but not dead
Invasive diastolic blood pressure (mmHg)	Vital sign	0.034	Exclude data with values below 0 and the value is 0 but not dead
Temperature (°C)	Vital sign	0.004	-
Breathe rate (bpm)	Vital sign	0.0002	Exclude data with values above 100 and the value is 0 but not dead
Oxygen concentration (%)	Vital sign	0.204	Set the values below 21 to 21. Exclude data with values above 100
Perfusion index	Vital sign	0.004	Exclude data with values above 50
CVP (mmHg)	Vital sign	0.250	Exclude data with values below or equal to 0
SPO_2_ (%)	Vital sign	0.001	Exclude data with values of 0 or greater than 100
Heart rate (bpm)	Vital sign	0.0002	Exclude data with values of 0 but not dead
White blood cell (×10^9^/L)	Laboratory examination	0.581	-
Neutrophilic granulocyte percentage (%)	Laboratory examination	0.583	Exclude data with values of 0
Hemoglobin (g/L)	Laboratory examination	0.581	-
Blood platelets (×10^9^/L)	Laboratory examination	0.581	-
Creatinine (mmol/L)	Laboratory examination	0.675	-
Total bilirubin (mmol/L)	Laboratory examination	0.725	-
pO_2_ (mmHg)	Blood gas	0.074	-
pCO_2_ (mmHg)	Blood gas	0.074	-
BE	Blood gas	0.096	-
pH	Blood gas	0.074	Exclude data with values below 6.7
Lactate (mmol/L)	Blood gas	0.074	Exclude data with values above 30
Gender	Demographics	-	-
Age (yrs)	Demographics	-	-
Weight (kg)	Demographics	-	-
bloc		-	-
Balance (mL)	Output volume-input volume	-	Exclude data with outputs or inputs below 0 or above 5,000 and empty values
Outcome	Dead or survived	-	-

### State, Action, and Rewards

Q-Learning is a basic form of Reinforcement Learning which uses Q-values (also called action values) to iteratively improve the behavior of the learning agent. Q-Learning technique is an off-Policy technique and uses the greedy approach to learn the Q-value. SARSA technique, on the other hand, is an On Policy and uses the action performed by the current policy to learn the Q-value. DQN model is based on Q-learning, which we use to uncover the relationship between states and actions, obtain new knowledge from existing data and find the best treatment plan to predict the reasonable range of fluid balance.

The purpose of reinforcement learning is to obtain an optimal policy for a specific problem to maximize the reward obtained under a given strategy. A strategy is mapped from state to action, which can determine what behavior to choose under a certain state and then enter the next state. The transition in the state is equivalent to adding a reward to the Markov decision process. Hence, the next state is related not only to the current state but also to the current action. Every decision consists of a status, behavior and reward. Given that the prediction is the interval of fluid balance, which can be divided into five intervals, we used the value iteration method of reinforcement learning.

In this research, each data point is a group of states, which includes 25 features except the balance and outcome. The action refers to how much liquid should be given under a certain state, which is divided into five groups by 0–20, 20–40, 40–60, 60–80, and 80–100% of the percentiles of fluid balance. Each group of actions and the corresponding balance is shown in Table 2. “Rewards” refers to a survival-derived score. At the terminal timestamp of each patient, we issued a reward of +15 if they survived their ICU stay and a reward of−15 if they died. The rewards were all 0 at the other timestamps.

**Table 2:** Fluid balance as the division of actions.

**Actions**	**Action 0**	**Action 1**	**Action 2**	**Action 3**	**Action 4**
Fluid balance intervals (mL)	< -110.68	−110.68 to −45.68	−45.68 to −0.67	−0.67 to 45.00	>45.00

### Algorithm Description

All the patients were divided randomly 8:2 into a training set and test set, corresponding to 2,095 (25,149 blocs) in the training data and 348 patients (6,326 blocs) in the test data. We needed to evaluate the expected reward when performing an action in a certain state; the higher the expected reward is, the better the effect of performing this action. The reward is called *Q*(*s, a*), with *s* being the current state and *a* being the performing action. *Q*(*s, a*) is also the q-value performing action *a* in state *s*. All *Q*(*s, a*) are initialized as 0, and *Q*′(*s, a*) is updated by performing different actions under different states and obtaining rewards from different next states. Then, the model can learn the possibilities of different *Q*(*s, a*) under a certain state and choose the action with the highest possibility as the next action.

There are two methods to update *Q*′(*s, a*): SARSA and Q-learning (10).

The formula for updating the *Q*′(*s, a*) in SARSA is as follows:


$$\begin{array}{l} Q^{\prime }\left(s,a\right)\leftarrow Q\left(s,a\right)+\alpha \left[r+\gamma Q\left(s^{\prime },a^{\prime }\right)-Q\left(s,a\right)\right]\\ \;s\leftarrow s^{\prime };a\leftarrow a^{\prime } \end{array}$$

The formula for updating the *Q*′(*s, a*) of Q-learning is as follows:


$$\begin{array}{l} Q\left(s,a\right)\leftarrow Q\left(s,a\right)+\alpha \left[r+\gamma max_{a^{\prime }}Q\left(s^{\prime },a^{\prime }\right)-Q\left(s,a\right)\right]\\ \;s\leftarrow s^{\prime } \end{array}$$

where r is the reward of the current action and α and γ are the parameters of 0–1.

When updating the *Q*′(*s, a*), SARSA chooses *a*′ from all possible next actions to the next state *s*′ and obtains *Q*′(*s*′, *a*′). Then, it performs *a*′ in state *s* to state *s*′. *Q*′(*s, a*) will be updated in the next iteration from one next action in state *s*′. Q-learning updates *Q*(*s, a*) with the highest *Q*′(*s*′, *a*′) in state *s*, but the next action will be chosen in another way in the next iteration rather than using the *a*′ of *Q*′(*s*′, *a*′). In general, the *a*′ in state *s* is chosen randomly; in this case, we could find out how much balance (*a*_1_) the patients have in state *s*_1_, then enter state *s*_2_, but if the action is random (such as *a*_2_), and all patients in state *s*_1_ did not have the balance *a*_2_ in the existing data, we would not know what the next state could be. Thus, the next state should be chosen from the given sequence of data. The updated strategy for *Q*(*s, a*) with SARSA is exactly the same as the actual implemented strategy, which is called on-policy, while inconsistent with Q-learning, which is called off-policy. SARSA is aimed at learning the characteristics of the original data, and Q-learning tends to discover new strategies (11).

### Data Evaluation

First, we used SARSA to learn the characteristics of the original data to then obtain the relationship between reward and mortality to evaluate whether the rewards were reasonable. *Q*(*s, a*) is the expected reward. The relationship between the expected reward and mortality could be acquired by calculating the mortality rate of each data point in *Q*(*s, a*). Ideally, the higher the expected reward is, the lower the mortality rate. We used the training set to build the SARSA model. Since the state cannot be exhaustive, we used a function (neural network) as the state to build a reinforcement learning model. The neural network consists of 1 input layer, two hidden layers, and one output layer. The input layer consists of 25 features (25 nodes). Each hidden layer has 128 hidden nodes. The output layer is the probability of *Q*(*s, a*) corresponding to the five action categories, and softmax is used to select the highest probability action as the final output.

Each node of the neural network represents a function and includes weights *w* and biases *b*. The weights are different in different nodes, and the biases are the same in the same layer and different in different layers. *w* and *b* are initialized as randomly generated decimals of a normal distribution with a mean value of 0 and a standard deviation of 1, generally within the interval [−1,1]. Due to the size of the training set, data are usually trained in small batches in a fixed-size sample that is randomly selected from all training data for each iteration. The sample size is called the batch size, and the batch size of this project is 32. First, the network receives the input and calculates using the received input and parameters, comparing the estimated output with the real output, it obtains the mean square error, and then it updates the parameters of each node according to the error. The learning rate for updating the parameters is 0.0001 to ensure that the amplitude of each update parameter will not be too large so that the current parameters can gradually approach the best parameters. Normally, the error will keep decreasing during training, and the decrease will be fast and then slow. When the error becomes stable as the training times increase, the training can be stopped to avoid overfitting.

**Figure 2:**
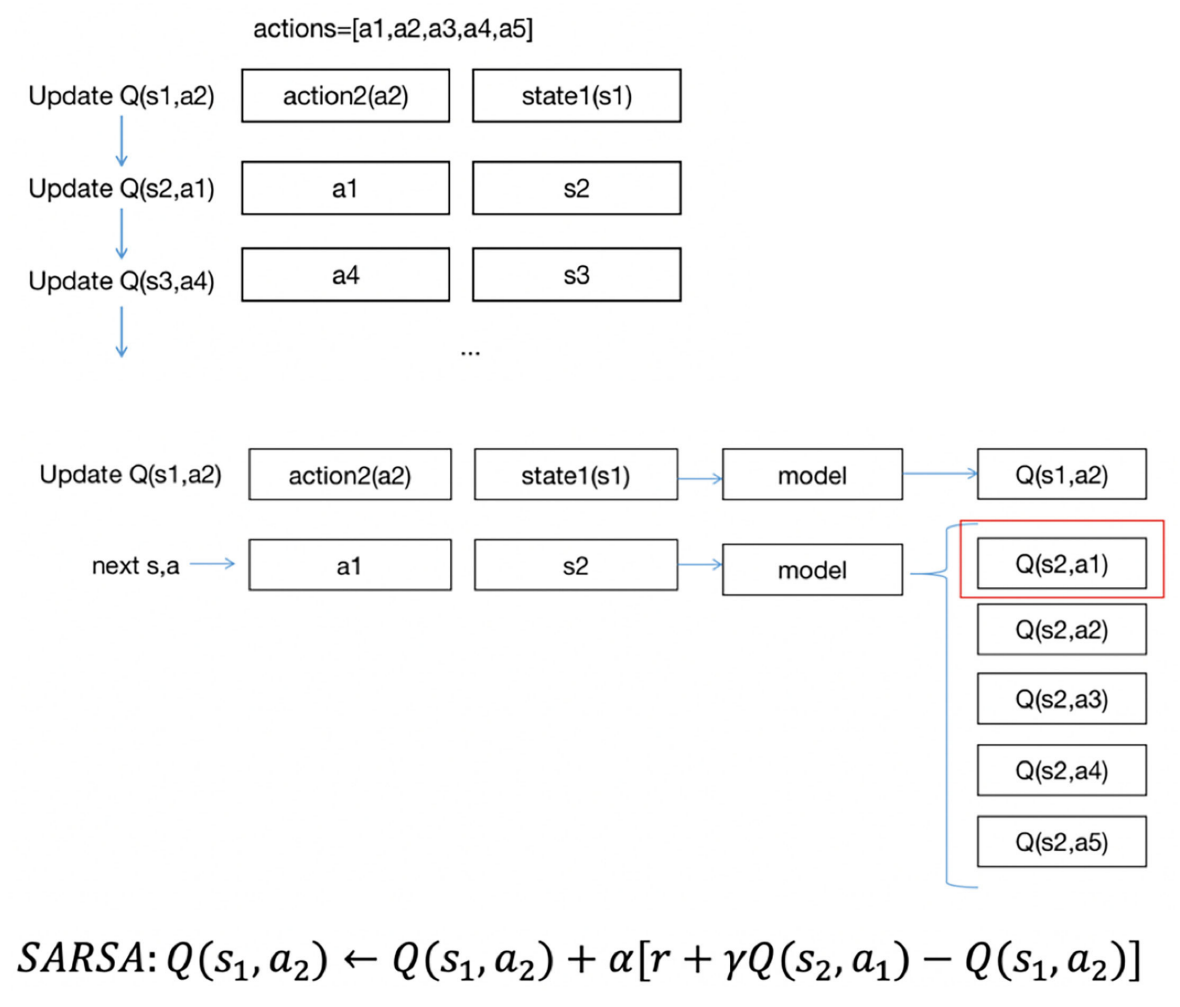
SARSA updates approach.

Figure 2 shows the approach of SARSA updates *Q*(*s, a*). In the project, there are five actions, *a*_1_, *a*_2_, *a*_3_, *a*_4_, *a*_5_. Suppose the states are *s*_1_,*s*_2_,*s*_3_.... Each data point includes a state and action group. The updating order is the same as the data order. As shown in Figure 2, suppose the first state is *s*_1_, the action to be performed is *a*_2_, and we update *Q*(*s*_1_, *a*_2_). With first input *s*_1_, we could have five Q values of five actions, then we could obtain *s*_2_ as the next state and *a*_1_ as the next action, using *Q*(*s*_2_, *a*_1_) to update *Q*(*s*_1_, *a*_2_). Then, we would update *Q*(*s*_2_, *a*_1_)… in the same way until the end of the iterations.

### Balance Prediction

Next, we used deep Q-learning (DQN) based on Q-learning to uncover the relationship between states and actions, obtain new knowledge from existing data and find the best treatment plan to predict the reasonable range of fluid balance. We used the training set to train the model and the test set to make predictions on the trained model.

**Figure 3:**
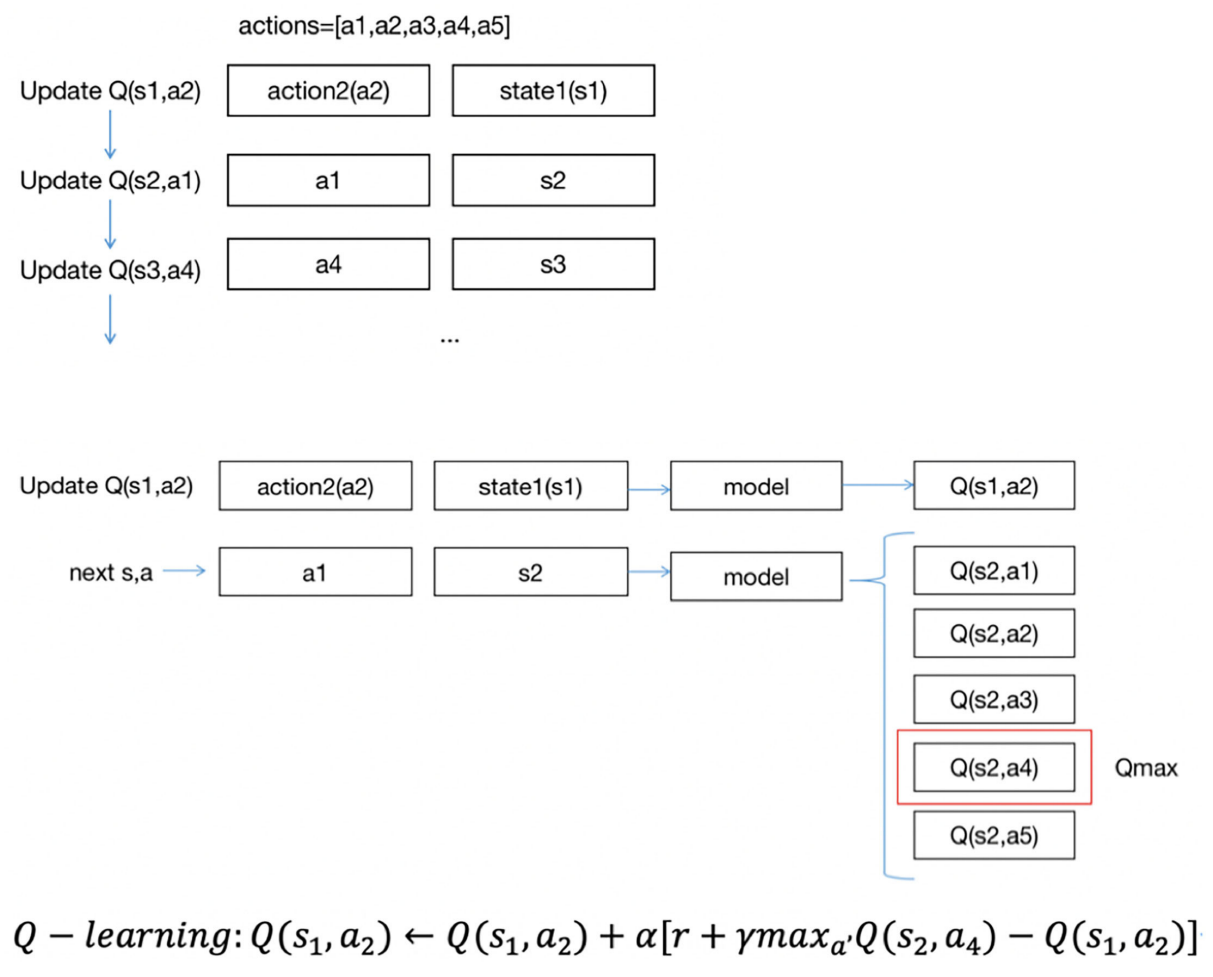
DQN updates approach.

Figure 3 describes the approach of DQN updates with *Q*(*s, a*). The updating order of *Q*(*s, a*) is also the same as the data order; unlike SARSA, DQN uses the highest *Q*(*s, a*) among five *Q*(*s, a*) to update *Q*(*s*_1_, *a*_2_). Suppose the highest *Q*(*s, a*) is *Q*(*s*_2_, *a*_4_), and we use *Q*(*s*_2_, *a*_4_) to update *Q*(*s*_1_, *a*_2_).

The principle of updating *Q*(*s, a*) with DQN and Q-learning is the same. Both use the max method. However, there will be an overestimation problem. If the data are noisy, the best *Q*(*s, a*) is not the largest *Q*(*s, a*). There may be biases after each iteration, and the biases will approach Q with greater values (12).

To solve the problem, we use double deep Q-learning (DDQN). The method of updating *Q*(*s, a*) with DDQN is as follows:


$$\begin{array}{l} Q\left(s_{t},a_{t}\right)\leftarrow Q\left(s_{t},a_{t}\right)+\alpha \left[R_{t+1}+\gamma Q^{\prime }\left(s_{t+1},a\right)-Q\left(s_{t},a_{t}\right)\right]\\ \;a=max_{a}Q\left(s_{t+1},a\right) \end{array}$$

**Figure 4:**
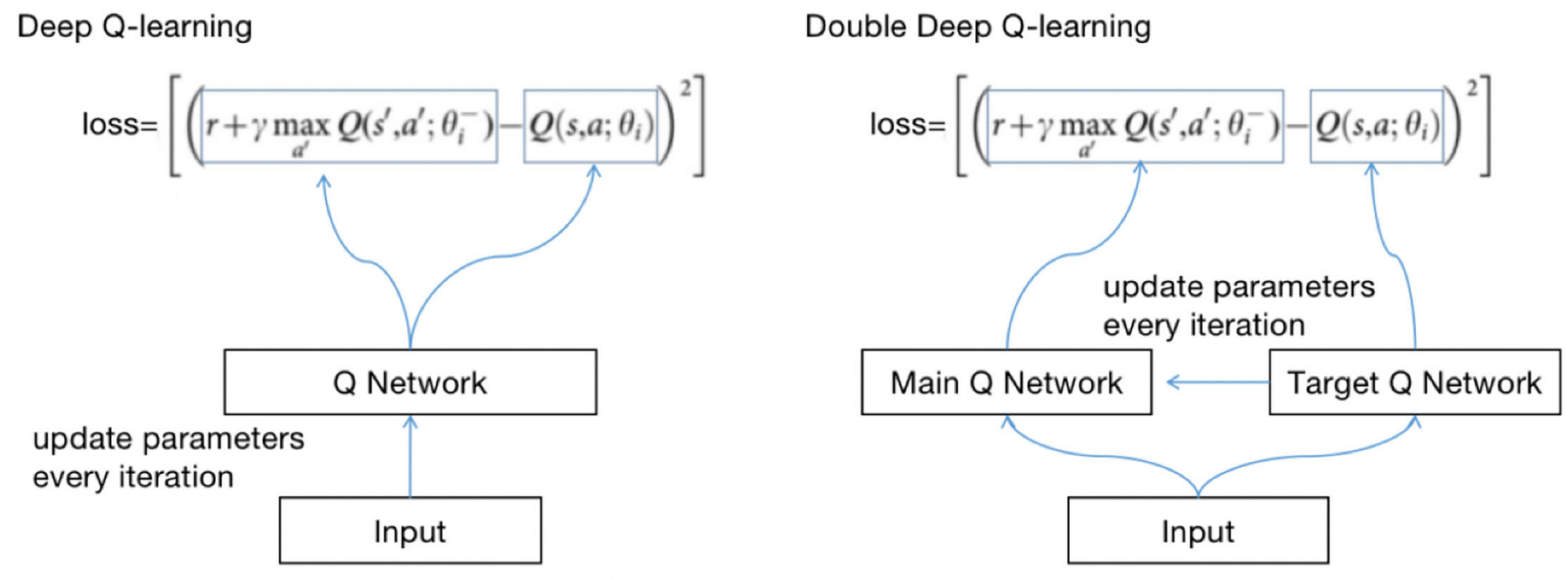
Calculating loss with DQN and DDQN.

The method of calculating losses with DQN and DDQN is shown in Figure 4.

θ is the parameter of the neural network; the method of obtaining Q is called selection, and the calculation loss is called an evaluation. DDQN implements the two methods with two Q networks, avoiding the risk of using the same Q network.

We define two identical structured Q networks, the primary QN and target QN, train with the primary QN and evaluate with the target QN (13). The calculation process is as follows:

(1) Use the primary QN to select the action with the highest *Q*(*s, a*) in *s*_*t*+1_, where *Q*(*s, a*) is called *q*_1_;
(2) Use target QN to evaluate the *Q*(*s, a*) of the action, and the evaluated *Q*(*s, a*) is called *q*_2_;
(3) Set the difference between *q*_2_ and *q*_1_ as the loss, update the parameters of the primary QN with back propagation, and do not update the parameters of target QN;
(4) Repeat (1) (2) (3) until convergence.

### Algorithm Evaluation

The algorithm evaluation method compares the original average reward and the average reward calculated by the model on the test set. If the average reward calculated by the model is higher than that of the original test set, we can argue that the model has an effect on optimizing treatments. However, because the model is built on the training set, we cannot ensure that the distribution (probability of occurrence) of the test set is the same as that of the training set, and it is inaccurate to use the mean prediction rewards for comparison. To ensure that the two groups of data are unbiased, the inverse probability score (IPS) is generally used for approximating the test set to the training set distribution.

The formula of the IPS is as follows:


(1) $$\begin{array}{l} V_{IPS}^{\pi }=\frac{1}{\left|N\right|}\sum_{i=\;1}^{N}\frac{A_{i}r_{i}}{\pi_{i}\left(x_{i}\right)} \end{array}$$

where A is whether an event occurs or not, r is the reward, and π_*i*_(*x*_*i*_) is the probability of an event occurring. If the probability on the test set is lower than that of the training set, as the denominator, *r* will be multiplied by a multiple greater than 1, which is the inverse. If the probability of an event occurring is correct, the test set will be unbiased. However, there are two problems. First, we cannot ensure that the probability is completely correct. Second, the variance will be too large if the probability is very small.

We can also use regression-based model *m*(*x*_*i*_) for prediction, and the formula is as follows:


(2) $$\begin{array}{l} m\left(x_{i}\right)=E\left(r|A=1,x\right) \end{array}$$

where A indicates whether AI policy is consistent with clinical policy, 1 indicates consistency and 0 indicates inconsistency.


(3) $$\begin{array}{l} V_{model}=\frac{1}{N}\sum_{i=1}^{N}\left[A_{i}r_{i}+\left(1-A_{i}\right)m\left(x_{i}\right)\right] \end{array}$$

If *A* = 1, (1 − *A*_*i*_) is 0, and according to the formula, the original reward will be used. If *A* = 0 and *A*_*i*_*r*_*i*_ is 0, the predicted reward will be used. Similarly, if the model is correct, the result will be unbiased.

A double-robust estimator is the combination of IPS and a regression model. The formula is as follows:


(4) $$\begin{array}{l} V_{DR}^{\pi }=\frac{1}{\left|N\right|}\sum_{i=1}^{N}\left[\frac{A_{i}\left(r_{i}-m\left(x_{i}\right)\right)}{\pi_{i}\left(x_{i}\right)}+m\left(x_{i}\right)\right] \end{array}$$

OR:


(5) $$\begin{array}{l} V_{DR}^{\pi }=\frac{1}{\left|N\right|}\sum_{i=1}^{N}\left[\frac{A_{i}r_{i}}{\pi_{i}\left(x_{i}\right)}-\frac{A_{i}-\pi_{i}\left(x_{i}\right)}{\pi_{i}\left(x_{i}\right)}m\left(x_{i}\right)\right] \end{array}$$

where *m*(*x*_*i*_) is the model, which is used for predicting the reward. If the model is correct, according to formula 4, the model is able to predict *r*_*i*_, and if *r*_*i*_ − *m*(*x*_*i*_) = 0, $V_{DR}^{\pi }=\frac{1}{\left|N\right|}m\left(x_{i}\right)$, the result is unbiased. If the model is not correct, the probability of an event occurring is correct. According to formula 5, $V_{DR}^{\pi }=\frac{1}{\left|N\right|}\overset{N}{\underset{i=1}{\sum }}\frac{A_{i}r_{i}}{\pi_{i}\left(x_{i}\right)}$, and the result is also unbiased. In other words, at least one of the models and the event probability estimation is correct, and the result is unbiased (doubly robust) (14–17).

### Model Validation

Patient data from July 2019 to April 2020 were extracted from PICMISD as the validation set. We compared the predictions and actual clinical conditions and prognoses of four different types of negative fluid balance and fluid infusion in the validation set. The preprocessing method of the validation set is the same as that used for the data collection and cleaning strategy in Section 2. Lastly, the validation set included 399 sepsis patients (representing 5269 time-blocks) were used for verification, with 357 survivors (including 4677 time-blocks), and 42 non-survivors (including 592 time-blocks). We divided the predicted and clinical 5-group fluid balance into two groups based on the total amount of fluid (positive or negative balance). The previous categories 0, 1, and 2 correspond to the new category 0, indicating negative fluid balance (negative balance), and categories 3 and 4 correspond to the new category 1, indicating fluid infusion (positive balance). In addition, the actual negative fluid balance and fluid infusion were determined according to the actual clinical PICMISD records. We calculated the morality of the four combinations of prediction and clinical results. The mortality rate is the lowest when the predicted fluid management strategy is the same as the actual strategy.

### Statistical Analysis

All statistical analyses were performed using SPSS Statistics for Windows (version 19.0, IBM Corporation, Armonk, NY) and R 3.4.3. Student's *t*-test or the Wilcoxon rank sum test was used to compare continuous variables. The categorical data were compared using the chi-square test or Fisher's exact test. The Kruskal-Wallis test was used to compare differences in continuous parametric variables with abnormal distributions. Differences in the variables between the groups were considered statistically significant at the a *p* level of <0.01.

## Results

### General Description

Table 3 shows the significant difference between each feature of the training set and test set. The *p*-values of all the features are greater than 0.01, which means that there is no significant difference between the two datasets.

**Table 3:** Comparison of 27 features of the training set and test set.

Features^*^	**Training set**	**Test set**	**Validation set**	***p-*value (training**	***p*-value (training vs**.
	**median (25th, 75th)**	**median (25th, 75th)**	**median (25th, 75th)**	**vs. test set)**	**validation set)**
Invasive mean pressure	88.33 (81.56–95.57)	89.25 (81.76–97.12)	83.74 (78.0–89.9)	0.1320	0.1320
Invasive systolic blood pressure	131.57 (120.47–143.62)	132.91 (121.83–145.56)	122.56 (112.02–135.0)	0.2992	0.2992
Invasive diastolic blood pressure	67.33 (60.75–74.22)	68.45 (61.41–76)	64.29 (58.79–69.58)	0.0144	0.0144
Temperature	37 (36.5–37.5)	37 (36.55–37.5)	37.0 (36.5–37.5)	0.2956	0.2956
Breathe	18.08 (15.86–20.83)	18.12 (15.71–21.1)	17.33 (15.38–20.2)	0.4522	0.4522
Oxygen concentration	31 (28–38.75)	31 (27.76–39.21)	36.29 (30.53–43.12)	0.1943	0.1943
Perfusion index	1.5 (0.79–2.4)	1.6 (0.81–2.63)	1.13 (0.64–1.87)	0.0224	0.0224
CVP	8 (6.5–9.64)	8 (6.33–9.61)	8.33 (7.0–10.0)	0.1711	0.1711
SPO_2_ (%)	98.64 (97.45–99.6)	98.38 (97.14–99.29)	98.4 (96.84–99.5)	0.0500	0.0500
Heart rate	92.86 (82.5–103.67)	93.45 (82.33–105)	94.56 (85.1–102.97)	0.1275	0.1275
White blood cell	11.8 (8.49–16.59)	11.22 (7.94–15.47)	11.61 (7.71–16.29)	0.2308	0.2308
Neutrophilic granulocyte percentage	86.1 (80.6–90.2)	86.2 (80.46–90.4)	88.05 (82.3–92.03)	0.0163	0.0163
Hemoglobin	96 (86–109)	97 (88–110)	91.0 (83.67–100.46)	0.0190	0.0190
Blood platelets	144 (89–208)	138 (80–208)	93.0 (63.67–140.92)	0.0660	0.0660
Creatinine	86 (60–138)	79 (55–139)	106.0 (79.0–164.0)	0.0221	0.0221
Total bilirubin	16.9 (11.4–30.9)	16.7 (11.2–30.1)	28.1 (15.3–59.49)	0.2970	0.2970
Lac	1.3 (0.9–1.83)	1.30 (1–2)	1.6 (1.13–2.59)	0.3278	0.3278
pO2	92.80 (79.3–111)	92.91 (79.5–110)	94.72 (79.26–117.0)	0.4295	0.4295
pCO2	39.05 (35.8–42.6)	39.3 (35.95–43.1)	39.85 (36.26–43.0)	0.3987	0.3987
BE	3.03 (0.4–5.47)	3.17 (0.6–5.9)	3.0 (0.0–5.6)	0.4494	0.4494
pH	7.45 (7.42–7.48)	7.45 (7.41–7.48)	7.44 (7.41–7.47)	0.3254	0.3254
Age	62 (48–70)	62 (50–70)	59.0 (50.0–68.0)	0.2671	0.2671
Weight	65 (58–75)	65 (58–75)	65.0 (60.0–74.0)	0.4209	0.4209
Bloc	8 (4–13)	8 (4–12)	9.0 (4.0–13.0)	0.1800	0.1800
Fluid balance	−20.83 (−90.66–32.87)	−19.24 (−90.25–37.31)	−31.19 (−109.05–36.81)	0.1606	0.1606

* *All parameters do not obey normal distribution*.

### Data Distribution

**Figure 5:**
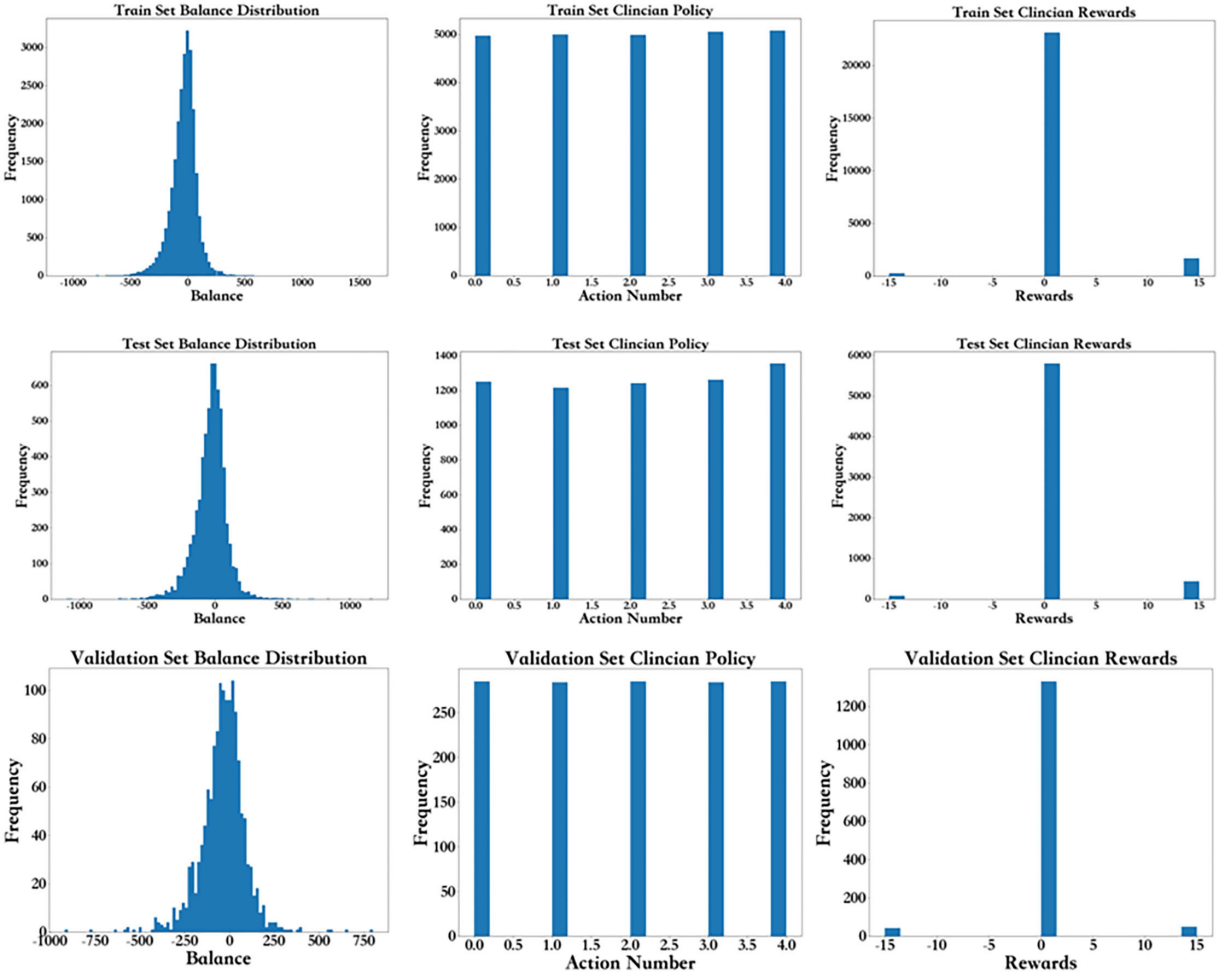
Frequency distribution for the balance distribution, action distribution, and reward distribution of the training set, test, and validation set.

As shown in Figure 5, from left to right, the first row shows the fluid balance distribution, action distribution and reward distribution of the training set based on the frequency. The test set shown in the second row follows the same distribution. Each data point represents the data from each period for each patient. The balance distribution figure shows that most patients had a balance of ±500, with the highest being 1608.92 and lowest being −1097.58, which was similar to those in the test set. The action distribution figure shows that although the balance of each patient for each period is mostly different, the percentage of each action in the training set and test set are basically the same. The number of each group of actions in the training set and test set are the same. In the reward distribution figure on the training set, the rewards of 2,095 individuals are alive, and 348 individuals died. In addition, most of the rewards were 0. The proportion of rewards in each part of the test set is basically the same as that of the training set. The results above show that the distributions of the training set, test set, and validation set are basically the same.

### Prediction of SARSA

**Figure 6:**
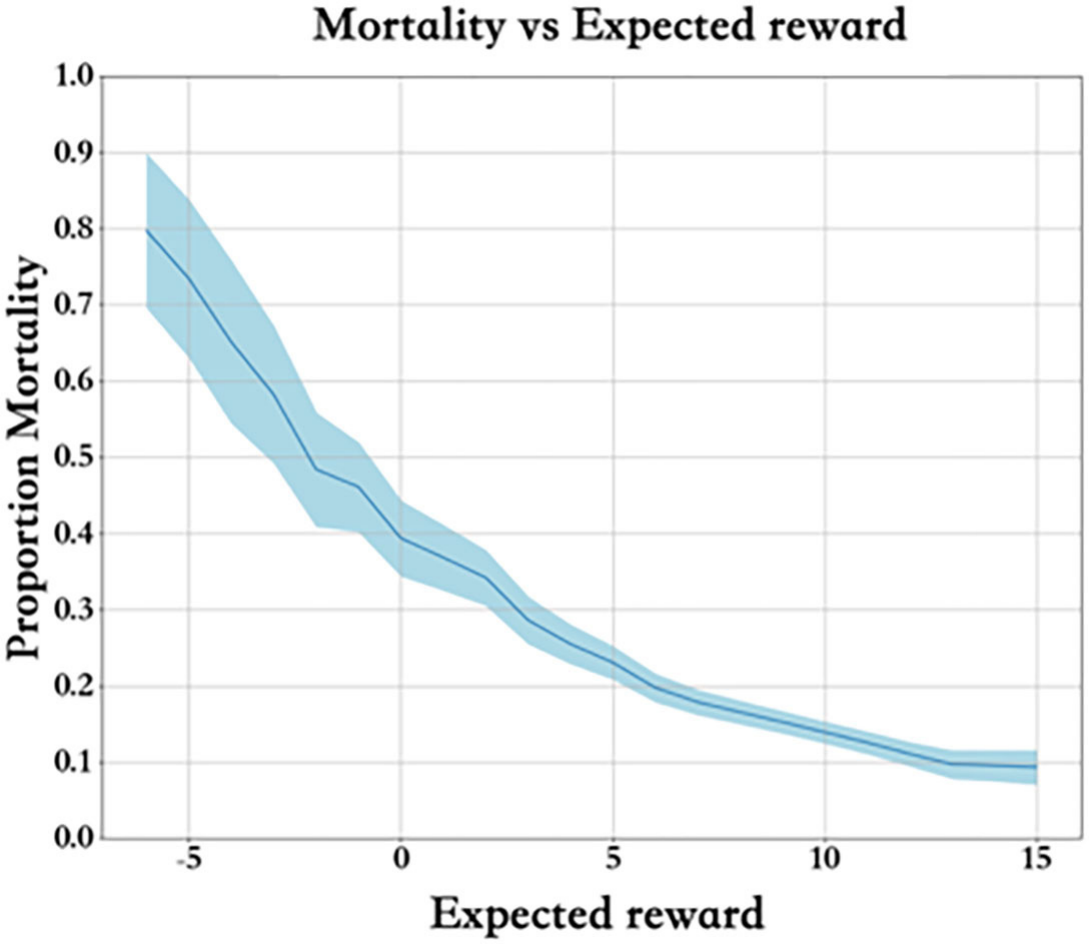
Mortality and expected reward.

Each training dataset includes a group of states and actions *Q*(*s, a*). Figure 6 describes the mortality and expected reward of the training set. Clearly, the higher the expected reward is, the lower the mortality rate, and the reward value is properly set.

### Prediction of Q-Learning

**Figure 7:**
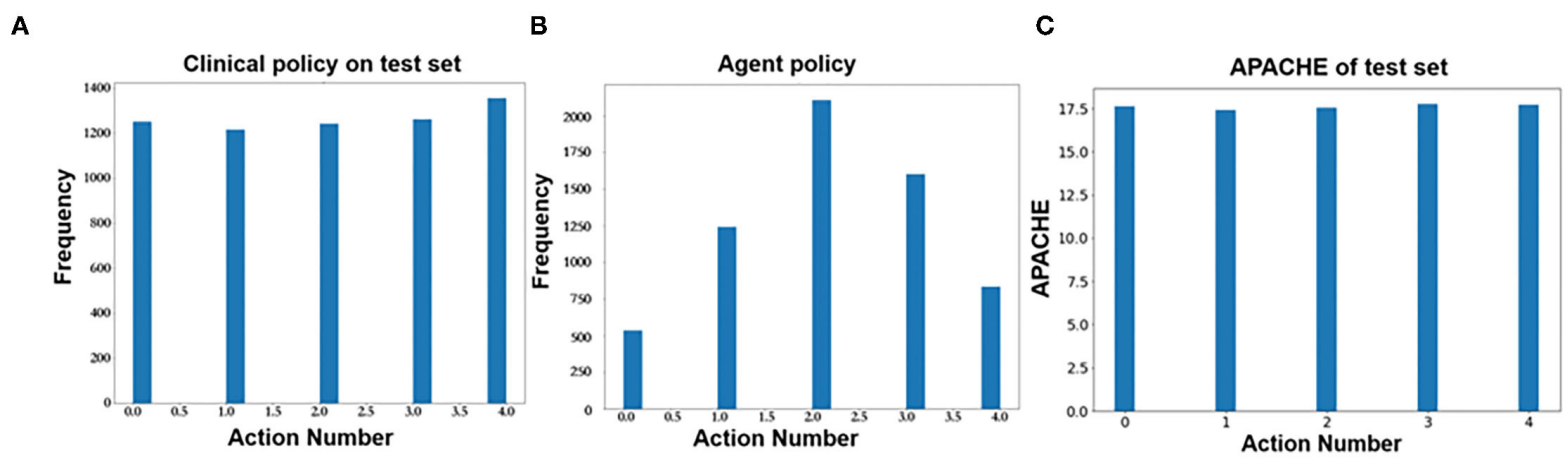
**(A)** Action distribution of clinical policy on the test set. **(B)** Action distribution of AI policy on the test set. **(C)** Action distribution of APACHE II on the test set.

Figure 7B shows the predicted action distribution of the test set. Action 2 has the highest amount among the five actions, and the corresponding balance is −45.68 to −0.67. Action 0 and action 4 have the lowest amount, which corresponds to too little and too much balance. In comparing the origin distribution in Figure 7A, the amount of balance that was too high and too low decreased significantly. Figure 7C shows the APACHE II score of these different actions.

**Figure 8:**
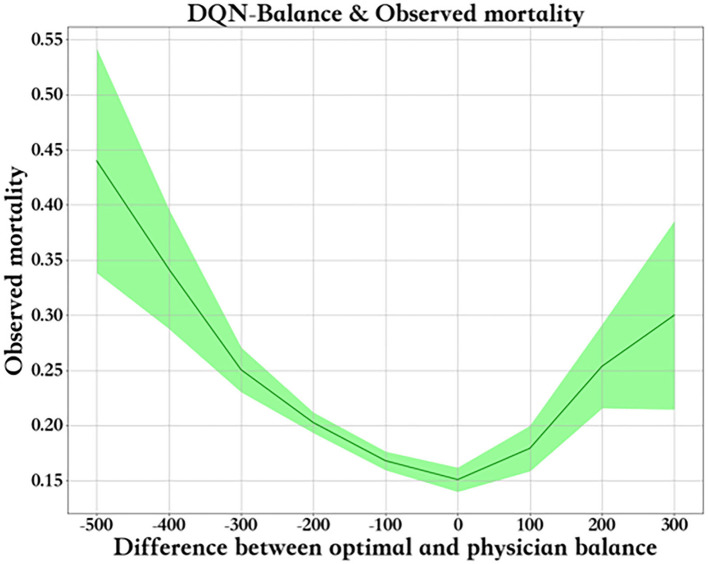
Predicted - real fluid balance difference and morality.

Figure 8 shows the relationship between fluid balance difference and mortality. We used the median fluid balance interval as the predicted value, and the difference was the predictive value minus the actual value. The closer the model is to the clinical fluid balance difference, the lower the mortality rate. This trend shows that the model is able to conclude clinical rules. However, a fluid balance difference that is too high or too low is not good for patients. Mortality is higher in patients with a lower fluid balance difference than in those with a higher fluid balance difference. The supplemental figure shows the predicted - real fluid balance difference and APACHE II score.

### Evaluation

A double-robust estimator was used to calculate the average expected reward [*Q*(*s, a*)] of the original test set, and the average predicted reward was calculated by the Q-learning model. The result is shown in Table 4. The results show that the average reward keeps increasing as the iterations increase. The increase is significant at the beginning, while the number of iterations rises from 20,000 to 30,000. Compared to the first 10,000 iterations, the increase in the average reward is much lower and could gradually become stable in future iterations. There could also be a risk of overfitting. Thus, we chose the model with 30,000 iterations as the final model.

**Table 4:** Average expected reward.

**Items**	**Average reward**
Original data from test set	4.07
Q-learning model (3,000 iterations)	4.05
Q-learning model (10,000 iterations)	9.06
Q-learning model (20,000 iterations)	10.37
Q-learning model (30,000 iterations)	10.47

### Validation

**Figure 9:**
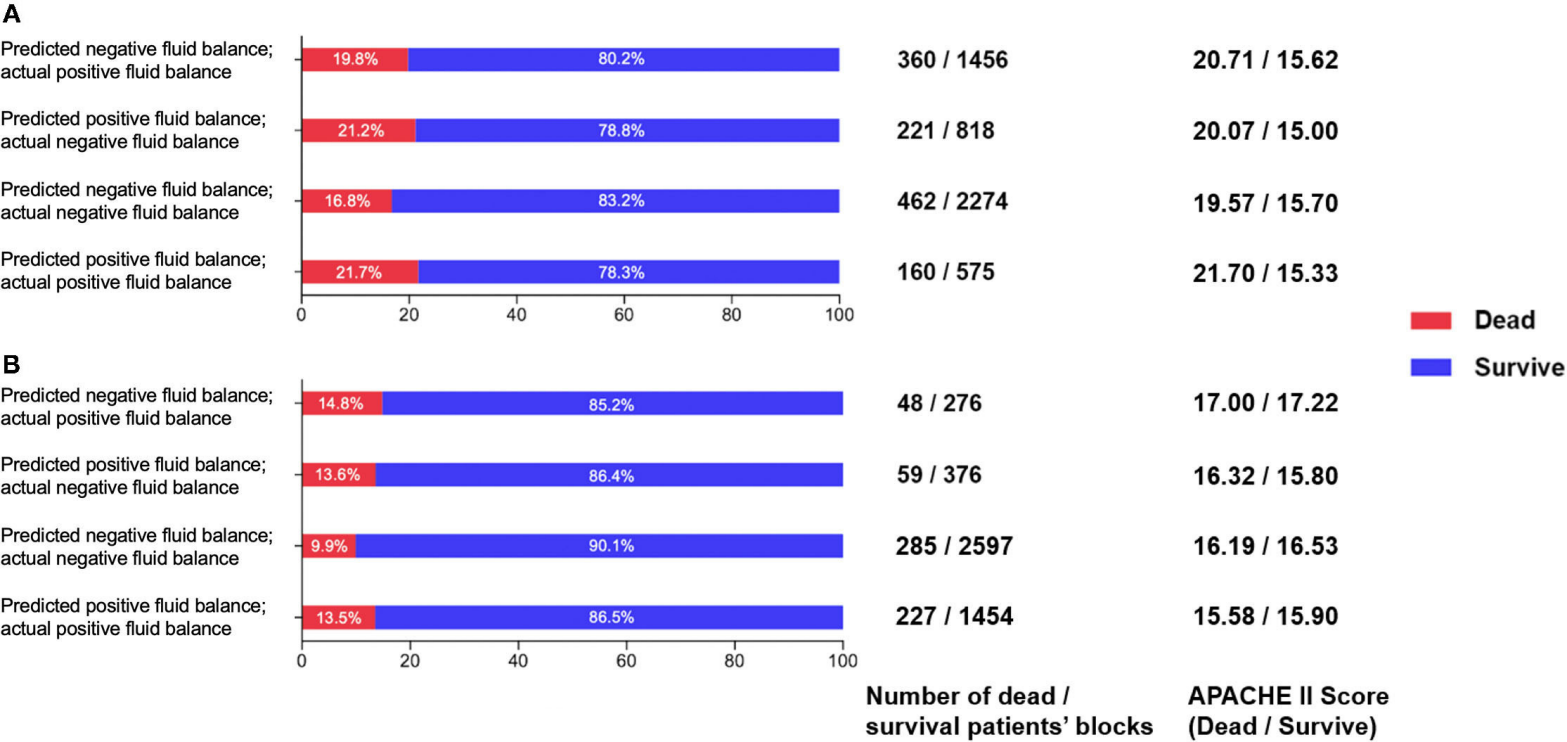
**(A)** Comparison of survival and death patients' blocks in the prediction and clinical balance groups in the validation set. **(B)** Comparison of survival and death patients' blocks predictions and clinical balance in the test set.

As shown in Figure 9A, patient mortality was lowest when negative fluid balance was predicted to be the same as clinical in both the validation set and the test set. The mortality is lower when the reinforcement strategy is the same as the clinical strategy. When the reinforcement strategy is different from the clinical strategy, it is more serious to predict negative fluid balance as fluid infusion than to predict fluid infusion as negative fluid balance, and the mortality of the former is lower than that of the latter. The results are shown in Figure 9B, which show the high value of the model in reality.

## Discussion

Our study indicates that the use of reinforcement learning methods can clearly predict a patient's future liquid treatment strategy. To our knowledge, clinical decision-making is a process that usually not gives immediate feedback. A certain treatment strategy may improve or worsen the state of illness. However, it does not mean the treatment prior to change of the illness is right or wrong. In clinical practice, a series of treatment procedures work together to accomplish the therapeutic effect. Hence, clinical decision-making in ICU is not a simple static classification problem. Instead, it is a dynamic process which we should learn through environmental rewards and punishments. Under this circumstance, reinforcement learning should be used to maximize rewards or achieve specific goals through learning strategies in the interactive process of the environment. The SARSA model can be used to simulate the equation between expected mortality and actual mortality during the liquid treatment process. The Q-learning model shows that as the model prediction and actual intake and output become closer, the mortality rate decreases. Additionally, if the intake and output are too high or too low which caused abnormal fluid imbalance, the mortality rate would be higher. Patients with higher positive fluid imbalance may have higher mortality than those with a higher negative fluid imbalance. We used a double-robust estimator to calculate the average expected reward of the test set in the Q-learning model after training 30,000 times as the final prediction model. Using a validation data set, the results suggest that if the model predicts that the patient should be dehydrated while the patient is dehydrated under actual treatment, the fatality rate is significantly lower compared with other circumstances. In particular, these data are from the first 108 h of entering the ICU ward, which suggests that the resuscitation is not a positive mass fluid resuscitation. This is worth thinking about for the clinicians.

Fluid overload is related to the prognosis of severely ill patients with septic shock and/or acute respiratory distress syndrome (ARDS). Excessive fluid load may lead to a vicious cycle in which interstitial edema causes organ dysfunction, leading to fluid accumulation, organ edema and dysfunction. All the potentially harmful consequences of fluid overload in different end-organ systems have an impact on patient morbidity and mortality. Therefore, although infusion is advocated in early resuscitation strategies, the side effects of inappropriate or excessive infusion are increasingly recognized by practitioners. Fluid therapy can be considered a double-edged sword. In 2000, a retrospective cohort study conducted by Alsousand et al. indicated that patients with a negative fluid balance for at least one day during the first 72 h of septic shock had a better prognosis (OR 5.0; 95% CI 2.30–10.9, *p* < 0.001) (18). In 2006, the FACTT study showed that although negative fluid balance had no effect on the mortality rate, it can significantly reduce the time of mechanical ventilation and the ICU stay in critically ill patients (19). Since 2006, more studies have focused on the relationship between fluid balance and mortality in sepsis, suggesting the disadvantages of positive fluid balance for patients with sepsis from the perspective of evidence-based medicine (1–4). A recent systematic review involving fluid balance and prognosis in critically ill children suggests that after initial resuscitation, these children may develop edema and progress to fluid overload. More evidence shows that fluid overload causes more complicated treatment and serious outcomes, which could increase morbidity and mortality (20). Therefore, when treating patients with sepsis, we should be alert to the “reinjury” caused by excessive volume load, and more methods should be used to identify and assist in decision-making about fluid therapy strategies. In particular, our research has found that early infusion does not require a large amount of fluid infusion for the ICU admission in the real world. If fluid therapy can be performed more appropriately and a negative balance can be reached earlier, patients will ultimately benefit. For critically ill patients, at the beginning of ICU admission, it is necessary to choose an assessment every 6 h. Regarding the liquid usage within these 6 h period, actual fluid balance rather than the actual amount of input and output is the core of the resuscitation treatment. Our study provided effective treatment decision-making recommendations that have good predictive performance based on fluid balance and each time interval. For example, patients with “recommended negative fluid balance but actual negative fluid balance” have a better prognosis, while those with “recommended negative fluid balance but actual positive fluid balance” have the worst prognosis.

The average infusion volume of patients with severe infection and septic shock on the first day of admission to the ICU is lower than recommended by the Surviving Sepsis Campaign bundle. An infusion of more than 5 L of fluid on the first day of admission to the ICU is associated with a significant increase in the risk of death and a significant increase in hospitalization costs (21). Vincent and De Backer recently proposed a conceptual model for managing the shock state. This model is aimed at fluid management during the treatment of critical illness. The treatment of shock is divided into four phases: (1) recovery phase: the goal is to achieve the lowest blood pressure level sufficient to maintain life; (2) optimum phase: the goal is to increase cardiac output to meet the requirements of the body; (3) stable phase: the goal of this phase is organ support and avoiding complications; and (4) de-escalation phase: patients in this stage should gradually leave the ICU intervention measures. This approach formally puts forward the necessity of liquid de-escalation therapy (22, 23). On this basis, Monnet et al. (24) discussed different fluid management strategies, including early goal-oriented fluid management, late conservative fluid management, and late goal-oriented fluid removal management. In addition, the “Four-D” (medication, Dosage, Duration and Degradation) concept of fluid therapy is expanded. When treating patients with septic shock, four stages of fluid therapy should be considered to answer these questions. Doctors should be fully aware of the proper time to start or stop intravenous infusion, when to start reverse resuscitation or actively drain fluid, and when to stop reverse resuscitation. However, it is difficult to give a clear standard to explain how patients should implement fluid therapy accurately. These abstract concepts are not conducive to clinical implementation. Sometimes the success or failure of a treatment depends on the doctor's own experience and understanding of related theories. Recent studies have shown that achieving a negative volume balance in the ICU is associated with a reduction in 90-day mortality. An earlier negative fluid balance is associated with a reduction in mortality. Each liter of negative fluid balance increases the mortality rate (20). This finding shows that fluid treatment, especially the identification of the de-escalation stage, is of great significance. Our model proposes this possibility both theoretically and practically. Using the Q-learning model can provide us with the direction of liquid therapy for clinical reference. Our model really brings the theoretical idea of fluid resuscitation back to the scale of clinical operability, with the help of computer reinforcement learning.

However, we should never forget avoiding excessive negative fluid balance during treatment. Our goal for negative fluid balance is to remove excess volume in the interstitial spaces. However, the volume in the circulation must be removed first. When the interstitial fluid resorption rate (plasma refilling rate) is sufficient to prevent hypovolemia, hypotension does not occur. Currently, we do not completely know the lowest fluid resorption rate that may prevent hypotension. Studies have shown that in patients with severe negative fluid balance, increased fluid intake and urine output are related to a decrease in hospital mortality. However, achieving a more negative fluid balance compared to a mild fluid balance is not associated with reduced mortality (25). In addition to giving certain liquid treatment strategies, our model suggests that excessive negative fluid balance and positive fluid balance also bring side effects. Our model gives us a better basis for making choices, which gave a boundary between negative fluid balance and positive fluid balance. This is the contribution of machine learning to precision medicine.

Several limitations should be mentioned in this study. This study uses a single-center database. The sample size and the treatment stereotype of the treatment center may weaken the universality of the model. In particular, the weights of 18 blocks for each patient were equal in this study. At present, a method to solve the time series problem has been presented (26, 27). Further study, including temporal evolution along blocks of the fluid balance, can be performed. In addition, the amount and timing of negative fluid balance and positive fluid balance in this model cannot be completely calculated. Our finding can give directions for fluid therapy dependent on model predictions. Hence, the results given by the model should be combined with the clinical results. Any current medical applications of artificial intelligence cannot replace physician's medical decision making. Third, several confounding factors may influence the result of analysis. For example, usage of vasopressors and variation of cardiac functions may contribute to outcome and fluid balance. Patient's severity of illness is not invariable during the treatment progresses. Moreover, the treatment of severe illness is very complicated. Although we considered the impact of disease severity when analyzing each bloc, it is impossible for us to update the value of the relevant scoring system every 6 h according to current data. The impact of any machine learning model on actual clinical conditions must be confirmed by BCT studies. Whether other interventions have also affected the process and conclusions of reinforcement learning, we may answer and solve them through more advanced methods if possible (28, 29).

## Conclusion

This study proves that the reality of fluid therapy for patients with sepsis in the early stage of ICU admission is that a large amount of fluid infusion may not be required. If it can be converted to the negative fluid balance resuscitation phase as soon as possible, the patient's prognosis is better. Reinforcement learning methods were used to propose a possible predictive model for guiding the fluid therapy of patients with sepsis in ICUs.Our study presents a methodological model for fluid therapy. It is believed that machine learning will ultimately assist in clinical decision-making regarding the fluid therapy of critically ill patients in the future.

## Data Availability Statement

The data analyzed in this study is subject to the following licenses/restrictions: This data was collected by Peking Union Medical College Hospital Intensive Care Medical Information System and Database (PICMISD), which is a protected database. If needed, we would like to disclose the data after the permission of Ethics Committee of Peking Union Medical College Hospital. Requests to access these datasets should be directed to LS, slx77@163.com.

## Ethics Statement

The studies involving human participants were reviewed and approved by Institutional Research and Ethics Committee of Peking Union Medical College Hospital. The patients/participants provided their written informed consent to participate in this study.

## Author Contributions

YLo, WZ, and BD takes responsibility for the integrity of the work as a whole. LS is responsible for the study design and conception and drafted the manuscript. YLi, MS, and SZ were responsible for the study design and conception and for the data management and statistical analysis. LS, SL, XZ, and LW drafted the manuscript. All authors revised the manuscript for content. All authors contributed to the article and approved the submitted version.

## Funding

This study was funded by CAMS Innovation Fund for Medical Sciences (CIFMS) from Chinese Academy of Medical Sciences (No. 2021-I2M-1-062 & 056), National Key R&D Program from Ministry of Science and Technology of the People's Republic of China (No. 2021YFC2500800), Beijing Nova Program from Beijing Municipal Science and Technology Commission (No. Z201100006820126), China Health Information and Health Care Big Data Association Severe Infection Analgesia and Sedation Big Data Special Fund (No. Z-2019-1-001), China International Medical Exchange Foundation Special Fund for Young and Middle-Aged Medical Research (No. Z-2018-35-1902), Excellence Program of Key Clinical Specialty of Beijing for critical care medicine in 2020 (ZK128001), and Beijing Municipal Science and Technology Commission (No. Z201100005520051).

## Conflict of Interest

LS, SL, XZ, LW, WZ, and YLo were employed by Peking Union Medical College Hospital. YLi, MS, and SZ were employed by DHC Software Co., Ltd. The remaining author declares that the research was conducted in the absence of any commercial or financial relationships that could be construed as a potential conflict of interest.

## Publisher's Note

All claims expressed in this article are solely those of the authors and do not necessarily represent those of their affiliated organizations, or those of the publisher, the editors and the reviewers. Any product that may be evaluated in this article, or claim that may be made by its manufacturer, is not guaranteed or endorsed by the publisher.
